# Tenascin-C: From Discovery to Structure-Function Relationships

**DOI:** 10.3389/fimmu.2020.611789

**Published:** 2020-11-26

**Authors:** Matthias Chiquet

**Affiliations:** Laboratory for Oral Molecular Biology, Department of Orthodontics and Dentofacial Orthopedics, University of Bern, Bern, Switzerland

**Keywords:** recombinant protein, cDNA sequencing, electron microscopy, monoclonal antibodies, extracellular matrix, fibronectin, tenascin-C, rotary shadowing

## Introduction: How to Isolate an ECM Protein in 1980

Forty years ago, the tremendous complexity of extracellular matrix (ECM) was still largely an uncharted area, mainly because many of its components could only be solubilized by denaturing agents. Known were just five types of collagens, elastin, a couple of proteoglycans, and a few ECM glycoproteins, among them fibronectin, thrombospondin-1, and laminin-111 (1). The best studied was fibronectin (2), which became notorious for promoting specific cell adhesion to collagens. In parallel, the search for yet undetected large ECM glycoproteins continued. In 1981-82, Carter (3) observed several novel glycoproteins in human fibroblast ECM extracts. Among them, "GP250" was shown to be distinct from fibronectin but resisted isolation. However, between 1983-85 several research groups independently discovered and characterized a similar ECM glycoprotein that later became known as tenascin-C (see below). Its subunits were comparable in size to fibronectin but instead of dimers formed large (>10^6^ kDa) disulfide-linked oligomers. In the following paragraphs, the history and context of the individual discoveries of tenascin-C is briefly recounted. I then describe how a combination of methods available at the time lead to a detailed structural model of tenascin-C. This was the basis for trying to assign functions to different parts of the molecule. For reasons outlined below, this turned out to be more difficult than for fibronectin.

## Discoveries of tenascin-C

### Glial Mesenchymal Extracellular Matrix (GMEM) Protein

Working on brain cancer, Bourdon et al. (4) aimed at finding a glioma-specific cell surface marker. They used the new tool of generating a monoclonal antibody (mAb) library, which they screened for mAbs binding specifically to U-251 glioma cells. One mAb, 81C6, also strongly reacted with the ECM of these cells. The antigen recognized by this mAb was detected in human glioblastoma but few other neural cancers, and was absent from normal human brain. The novel ECM component was also present in restricted mesenchymal areas of normal adult tissues. Interestingly, “glial mesenchymal extracellular matrix” (GMEM) antigen was practically absent from normal skin, but was strongly expressed by fibrosarcomas and human fibroblast lines in culture. By radioimmunoassays, mAb 81C6 did not react with plasma fibronectin, collagen types I-V, and glycosaminoglycans. Immunoprecipitation of radiolabeled U-251 cell extracts revealed two bands of 230 and 210 kDa on reducing SDS gels. The authors concluded to have identified a novel human ECM protein of glial and mesenchymal origin that differed from fibronectin and any other ECM component known at the time.

### Myotendinous Antigen

Tenascin-C was discovered a second time due to its appearance in the developing vertebrate musculoskeletal system. Chiquet and Fambrough (5) aimed at finding mesenchymal ECM components that connect muscle fibers to tendons at the myotendinous junctions. Again a mAb library was generated, this time against chick skeletal muscle ECM, and screened for antibodies that specifically labeled tendons and myotendinous junctions in embryonic and adult limbs. The most promising, mAb M1, recognized a “myotendinous antigen” that was also present in restricted areas of a few other developing organs. After immunoprecipitation, a major band at 220 kDa and two minor (splice) variants at 200 and 190 kDa were observed. Despite of similar size, there were clear differences to fibronectin. First, proteolytic cleavage patterns of myotendinous antigen were distinct from fibronectin. Second, on non-reducing SDS gels the antigen had an Mr of >10^6^, indicating that it was a disulfide-linked oligomer instead of a dimer. Third, contrary to fibronectin, myotendinous antigen did not bind to gelatin but co-purified with proteoglycan, pointing to functional differences (6). In conclusion, a novel ECM protein presumably involved in muscle-tendon interactions was isolated.

### Hexabrachion

Erickson and Inglesias (7) investigated cell surface fibronectin preparations after rotary shadowing in the electron microscope (EM). The majority of particles in their samples had the typical V-shape of fibronectin dimers, but in addition they found molecules of a peculiar six-armed, gnat-like appearance that they called “hexabrachions”. In contrast to the rather uniform fibronectin subunits, hexabrachion arms showed distinct structural features. The authors therefore concluded that hexabrachions were not higher-order fibronectin oligomers, but represented a novel ECM protein contaminating fibronectin preparations.

### J1 Glycoproteins

The mAbs L2 and HNK-1 recognize a specific oligosaccharide epitope present on certain neural adhesion molecules. To identify unknown neural components carrying this epitope, Kruse et al. (8) used a mAb L2 column to enrich for L2/HNK-1 positive proteins from mouse brain. After removing N-CAM, L1, and MAG, they produced an antiserum against the “rest L2 fraction”. Interestingly, this antiserum inhibited neuron-astrocyte interactions in culture. On immunoblots, it reacted with a mixture of ECM proteins present in brain. According to their size in kDa, these proteins were called “J1-200/220” and “J1-160/180”, respectively. Later, collaborations established that J1-200/220 was identical to tenascin-C (9), and J1-160/180 to its paralog tenascin-R (10).

### Cytotactin

Grumet et al. (11) published a similar approach to isolate “cytotactin” from embryonic chicken brain. They purified the protein from a crude fraction of HNK-1-positive components by ion exchange chromatography, and showed it to be a disulfide-linked complex of 220, 200 and 190 kDa polypeptides like myotendinous antigen. Purified cytotactin blocked the activity of an antiserum that inhibited the adhesion of neurons to glial cells, again indicating that the novel ECM protein was involved in the interaction between the two cell types. Grumet et al. (11) also showed the distribution of cytotactin in the chick embryo, which agreed with the results for myotendinous antigen (5).

### Tenascin-C

Exchange of reagents and collaborations quickly established that the novel ECM protein described independently was in fact one molecular species (12). Eventually, the new name “tenascin” coined by Chiquet-Ehrismann et al. (13) became generally accepted. After discovery of the additional family members tenascin-R (14), -X (15), and -W/N (16), the original tenascin was amended to tenascin-C (17).

## Towards a Structural Model of Tenascin-C

**Figure 1:**
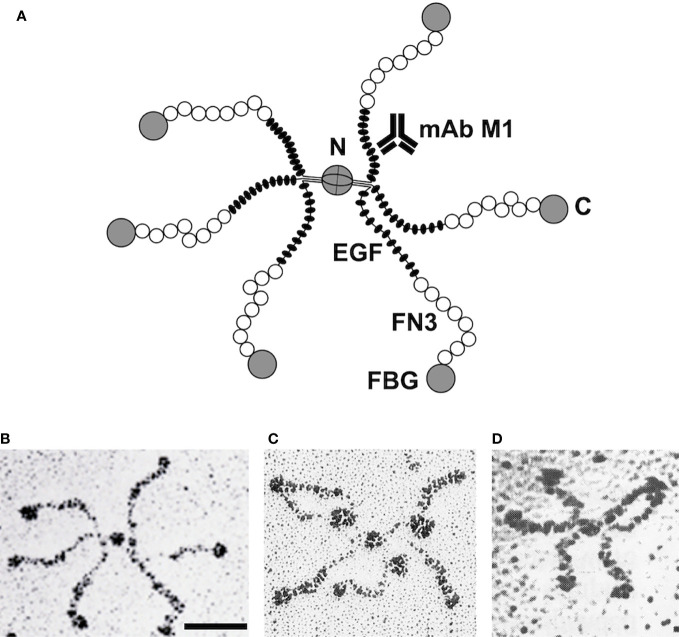
Correlation of tenascin-C domain structure with electron micrographic images. Model of the hexameric tenascin-C molecule derived from cDNA sequencing. Each arm corresponds to one subunit: two triplets of arms are connected by disulfide bridges at the central globular domain. C, C-terminus; N, N-terminus; EGF, EGF-like domains; FN3, fibronectin type III repeats; FBG, fibrinogen-like domain; mAb M1, monoclonal antibody binding site **(A)**. EM image of a single tenascin-C (hexabrachion) molecule after rotary shadowing. Bar, 50 nm **(B)**. EM image of tenascin-C molecule with three mAb M1 particles binding to the inner rod domain (EGF repeats) of its arms **(C)**. Recombinant tenascin-C mutant with a deletion of the EGF-like repeats. Note that the arms are shortened because their inner rod domain is missing **(D)**. Original micrographs **(B, C)** from Chiquet-Ehrismann et al. (18), and **(D)** from Fischer et al. (19) with permission.

A crude model of tenascin-C was already laid out in the first publications. The molecule consisted of several similar or identical 220 kDa subunits that were linked together at one end by disulfide bridges (6). EM images of rotary-shadowed molecules revealed an intriguing hexabrachion structure (**Figure 1**). From opposite sides of a central globule, two triplets of arms were emanating that showed distinct features. Each arm had a thin proximal rod fused to a thicker, flexible middle region and ended in a distal globular domain (7, 18, 19). From expression cloning using the new antibodies against tenascin/cytotactin, its first cDNA sequences were published in 1988 (20, 21). Fortunately, the tenascin-C cDNA contained sequence repeats coding for some small protein modules for which X-ray structures were already available. These were (from N- to C-terminus) an alpha-helical coiled coil domain suitable for oligomerization, epidermal growth factor (EGF)-like modules, a series of fibronectin type III (FN3) domains, and a single globular domain related to the fibrinogen gamma-chain (**Figure 1A**).

It was tempting to correlate the tenascin-C cDNA sequence with the structural features of hexabrachion particles as observed by EM. The N-terminal coiled-coil domain with adjacent cysteines could link two triplets of subunits at the central globe via disulfide bridges. The EGF-like repeats might correspond to the proximal rod domain of each hexabrachion arm, the stretch of FN3 domains to the flexible middle part, and the fibrinogen-like domain to the distal globule (**Figure 1A**). To prove this hypothesis for avian tenascin-C, two approaches were used. First, a library of monoclonal antibodies was generated against the purified molecule (18). Individual antibodies were tested for their binding to different recombinant tenascin-C fragments that were derived from various parts of the sequence (22). Simultaneously, individual mAbs were incubated with intact tenascin-C, and the mixture was examined in the EM after rotary shadowing. IgG molecules attached to hexabrachion particles could easily be identified and their binding sites mapped from such images (**Figure 1C**). For example, mAb M1 reacted with a recombinant chick tenascin-C fragment containing the N-terminal EGF-like repeats, and mAb Tn68 with a fragment comprising the C-terminal FN3 repeats (22). On EM images, mAb M1 bound to the proximal thin rod region of tenascin-C particles (**Figure 1C**), and mAb Tn68 to the flexible middle region close to the distal globe. Similarly, mAbs against other parts of the molecule were mapped by EM on intact tenascin-C.

Later, a complementary approach derived from analyzing recombinant deletion variants of full-length tenascin-C by EM. Fischer et al. (19) expressed and purified chick tenascin-C subunits that lacked specific parts of the intact molecule, but still assembled correctly into hexabrachion particles. Reactivity of such tenascin-C variants with antibodies matched with the specific deletions. A variant missing the EGF-like domains reacted with all tenascin-C specific mAbs except mAb M1, and when examined in the EM, it lacked the proximal rod-like part of the hexabrachion arms (**Figure 1D**). A FN3 deletion variant did not react with mAb Tn68, and missed the flexible middle region from the hexabrachion structure. Similarly, when the fibrinogen-like domain was deleted, hexabrachions without the distal globe of the arms were observed by EM. By these means, it was possible to generate a precise model of the hexameric chick tenascin-C molecule (19, 22) (**Figure 1**). Knowing the X-ray structures of the various modules from those of the original proteins, on paper the model of 1.3x10^6^ kDa hexameric tenascin-C could be extrapolated essentially to atomic resolution.

## Discussion: The Tricky Path From Structure to Function

Elucidation of the structure of a complex protein is rightly considered the basis for understanding its interactions and functions. In the ECM field in the early 1980s, fibronectin was the role model. Once its general structure was known, various defined functions could be assigned to distinct parts of the molecule, among them cell/integrin, gelatin/collagen, and heparin/glycosaminoglycan binding sites. In fibronectin, all these sites fully retained their individual functions as small proteolytic or recombinant fragments. Fibronectin appeared like an interface with independent functional units plugged in, enabling it to connect cells with ECM (23). However, the example of fibronectin spoiled us. For other large ECM proteins, the structure-function relationships turned out to be much more complex, and tenascin-C is a puzzling example. It has been classified as an adhesion-modulating “matricellular” protein with seemingly context-dependent activities. On the one hand, it is a poor adhesion molecule for many cells and notorious for inhibiting spreading of fibroblasts on fibronectin (18). On the other hand, it binds proteoglycans and promotes neurite outgrowth [for review, see (24)]. However, it proved difficult to assign these functions to specific parts of the molecule. Partially conflicting results were reported from using small recombinant tenascin-C fragments (22, 25, 26). In a reciprocal approach, Fischer et al. (19) instead analyzed avian tenascin-C deletion mutants for function. In this case, tenascin-C lacking the C-terminal fibrinogen-like domain completely lost its ability to inhibit fibroblast spreading. However, deletion of other regions (primarily adjacent FN3 repeats) diminished this activity as well. Similarly, the neurite-promoting activity of full-length tenascin-C disappeared completely by removing just the fibrinogen globe. However, when the FN3 repeats were omitted in addition, a strong neurite-promoting activity became unmasked in the EGF-like region, which was not seen with intact tenascin-C and did not depend on the fibrinogen domain. Thus in contrast to fibronectin, distinct activities of tenascin-C appear to depend on strong crosstalks between its various domains (19). Such interactions can also affect functions (such as glycosaminoglycan binding) that are observed with certain fragments but hidden in the full-length molecule (27). Intact tenascin-C is clearly much more than just the sum of its parts, which complicates the analysis of structure-function relationships. This should be taken into account when studying the more recently discovered roles of tenascins in the immune system (28), which are the topic of this special issue.

## Author Contributions

MC designed the article, assembled the literature, drafted and edited the manuscript, and prepared the figure.

## Funding

Own work on tenascin-C was supported by a postdoctoral fellowship of the Bay Foundation, and by a career development award (START fellowship #9060) and research grants #9474, #31007, #45952, #55551, #67029, and #107515 from the Swiss National Science Foundation.

## Conflict of Interest

The author declares that the research was conducted in the absence of any commercial or financial relationships that could be construed as a potential conflict of interest.
